# The efficacy and safety of lecanemab 10 mg/kg biweekly compared to a placebo in patients with Alzheimer’s disease: a systematic review and meta-analysis of randomized controlled trials

**DOI:** 10.1007/s10072-024-07477-w

**Published:** 2024-04-03

**Authors:** Karim Abdelazim, Ahmed A. Allam, Badreldin Afifi, Hebatullah Abdulazeem, Ahmed I. Elbehiry

**Affiliations:** 1 Department of Biotechnology, Institute of Graduate Studies and Research, Alexandria University, Alexandria, Egypt; 2 Department of Biology, Biotechnology Program, The American University in Cairo, New Cairo, Egypt; 3 Department of Molecular Biology and Genetics, Faculty of Arts and Sciences, Yıldız Technical University, 34220 Istanbul, Turkey; 4 Chair of Epidemiology, TUM School of Medicine and Health, Technical University of Munich, Munich, Germany; 5 Department of General Medicine, International Faculty, Russian National Research Medical University Named After NI Pirogov, Moscow, Russian Federation

**Keywords:** Lecanemab, Alzheimer’s disease, Monoclonal antibody, Adverse events, Amyloid-beta plaques

## Abstract

Alzheimer’s disease, prevalent in individuals aged 60 and above, constitutes most dementia cases and significantly impairs memory and cognitive functions. With global Alzheimer’s cases projected to triple by 2050, there is a pressing need for effective interventions. Lecanemab, a monoclonal antibody targeting amyloid-beta plaques, shows promise in slowing Alzheimer’s progression. Positive clinical trial results have instilled hope in patients, prompting ongoing research to advance understanding and intervention possibilities. To contribute to this knowledge base, we conducted a systematic review and meta-analysis, focusing on lecanemab’s efficacy and safety at a dosage of 10 mg/kg. This comprehensive approach aimed to address gaps in the current literature, scrutinize research disparities, and guide future investigations. Applying strict inclusion/exclusion criteria, we assessed study details, participant information, and intervention specifics, using the Cochrane risk of bias tool for quality evaluation. Statistical analyses, conducted with R software, included risk ratios and mean differences, assessing heterogeneity and publication bias. The meta-analysis reveals a significant positive effect of lecanemab (10 mg/kg biweekly) on cognitive outcomes in Alzheimer’s disease. Consistent reductions in ADCOMS, CDR-SB, and ADAS-cog14 scores across studies indicate drug efficacy with narrow confidence intervals and no significant heterogeneity. While TEAE shows no significant difference, heightened risks of ARIA-E and ARIA-H associated with lecanemab underscore the need for vigilant safety monitoring in clinical practice. Despite the drug efficacy, the study emphasizes a balanced assessment of benefits and potential risks associated with lecanemab, providing critical insights for clinicians evaluating its use in addressing cognitive impairment in individuals with Alzheimer’s disease.

**Supplementary Information:**

The online version contains supplementary material available at 10.1007/s10072-024-07477-w.

## Introduction

Alzheimer’s disease (AD) is a neurodegenerative disorder causing memory and cognitive decline, significantly impacting the quality of life, especially in individuals aged 60 and older, constituting approximately 70% of dementia cases [1, 2]. According to the Alzheimer’s Association, approximately a third of elderly people die with AD or similar dementia. The global prevalence of AD is expected to triple by 2050, reaching over 100 million cases, with variations attributed to demographics, age, and diagnostic criteria [2]. Notably, AD incidence increased by 40% in Umea, Sweden, from 2001 to 2006, underscoring the need for effective interventions [3].

Lecanemab, a monoclonal antibody, aims to slow AD progression and improve cognitive function by targeting Aβ plaques, a hallmark feature of AD pathology. Its mechanism involves binding to aggregated forms of the protein, promoting clearance from the brain, and reducing amyloid-beta burden [4, 5]. By targeting the toxic protofibrils of amyloid-β, lecanemab may have a disease-modifying effect that slows down the progression of cognitive and functional deficits in AD patients. This can help improve the quality of life for patients with mild AD and their families. However, it is not yet known whether the medicine helps in other ways such as slowing the development of AD in people without symptoms of memory loss [6–8].

Several clinical trials have produced positive findings about lecanemab, also known by its generic name BAN2401, regarding its safety and possible efficacy [4, 5]. There is ongoing research for the development of lecanemab to target the roots of AD, which may offer new hope for AD patients [9]. Use of lecanemab in older, less healthy, less well-educated, and more diverse populations, who are not included in previous clinical trials, may result in efficacy and safety outcomes that differ from those observed in trials [10].

The Food and Drug Administration (FDA) accelerated approval for lecanemab was granted in January 2023, acknowledging its potential in a population not included in previous clinical trials Despite promising phase III data indicating a 27% reduction in cognitive decline over 18 months, the FDA decision was based on phase II results, emphasizing the need for further research and caution in its use, currently restricted to individuals with mild cognitive impairment consistent with the clinical trial group [11].

In a study on lecanemab use compared to placebo in early Alzheimer’s, 1795 participants were enrolled, half of them received lecanemab. Results showed reduced amyloid markers and a slower cognitive decline over 18 months, but adverse events necessitate more extensive trials [12]. In the lecanemab Study 201 Core, a double-blind trial with 856 patients, 10 mg/kg of lecanemab biweekly significantly reduced amyloid plaques, slowed clinical decline over 12–18 months, and highlighted potential disease-modifying effects [6].

Our systemic review attempted to reduce bias with the use of explicit methods to perform a comprehensive literature search and critical appraisal of individual studies. Meta-analyses were aimed to assess the strength of evidence present on the lecanemab 10 mg/kg efficacy and safety. For efficacy, one aim was to determine whether an effect exists; another aim was to determine whether the effect is positive or negative and, ideally, to obtain a single summary estimate of the effect. For safety, the goal was to determine the presence of potential harm and quantify their risk, ideally providing a single summary estimate of safety concerns.

## Material and methods

### Search strategy

We conducted a comprehensive search across electronic databases, including PubMed, Scopus, Web of Science, Cochrane Library, and Clinical Trials.gov for publication period ranging from 2010 to 2023. The initial clinical trial on lecanemab (NCT01230853) took place in 2010 [13]. The search strategy used was “lecanemab OR leqembi OR BAN2401 OR BAN 2401 OR BAN-2401 AND Alzheimer’s disease OR Acute Confusional Senile Dementia OR Presenile Dementia OR Senile Dementia OR Alzheimer dementia (AD) OR Alzheimer sclerosis OR Alzheimer syndrome OR Alzheimer-type dementia OR ATD OR DAT OR Familial Alzheimer disease OR FAD” and their association with various Alzheimer’s disease-related terms, with a language constraint of English. This limitation was imposed due to language restrictions.

Our systematic review and meta-analysis specifically focused on peer-reviewed randomized controlled trials (RCTs) for several reasons. RCTs are widely acknowledged as the gold standard for establishing causality in research due to their randomized nature, which effectively controls for confounding variables and allows us to confidently attribute observed outcomes to the intervention being studied. The inherent design of RCTs also contributes to high internal validity by minimizing biases and ensuring that baseline characteristics are evenly distributed among intervention and control groups. Given that our primary objective is to assess the comparative effectiveness of interventions, RCTs facilitate a rigorous comparison between different interventions or between intervention and control groups. Moreover, RCTs hold a prominent position in the evidence hierarchy, providing robust and reliable evidence for clinical decision-making. While we recognize the potential value of other study designs, our deliberate choice to focus on RCTs aligns with our commitment to ensure the highest methodological rigor and internal validity in this systematic review.

### Study selection

We included peer-reviewed RCTs conducted in English that compare lecanemab at a dosage of 10 mg/kg biweekly against a placebo in AD patients. Included studies reported outcomes related to Alzheimer’s Disease Composite Score (ADCOMS), Alzheimer’s Disease Assessment Scale-Cognitive Subscale 14-item version (ADAS-Cog14), and Clinical Dementia Rating Scale-Sum of Boxes (CDR-SB).

In this study, the inclusion criteria encompass patients diagnosed with Alzheimer’s disease, with a specific focus on assessing the efficacy and safety of lecanemab in comparison to a placebo through peer-reviewed RCTs. There are no restrictions on age, gender, location, or ethnicity, allowing for a comprehensive examination across diverse patient groups. To maintain precision and reliability, the exclusion criteria involve the exclusion of animal studies, in vitro investigations, observational or laboratory studies, and research with redundant findings. Additionally, abstract-only presentations, reviews, books, posters, theses, editorials, notes, letters, case reports, case series, and conference papers are excluded, ensuring a stringent focus on peer-reviewed RCTs in this thorough analysis (Table 1). Studies were selected based on the inclusion and exclusion criteria by two independent authors (K.A., B.A) and cross-checked by K.A. and A.A.A. Any disagreement was settled by consensus among all authors.

**Table 1:** Inclusion and exclusion criteria list and their justification

Inclusion criteria	Justification	Exclusion criteria	Justification
1. Population: patients with Alzheimer’s disease	The primary focus is on individuals diagnosed with Alzheimer’s disease to ensure the relevance of the studies to the specified population of interest	1. Animal studies	Animal studies are excluded as they may not accurately represent the response of human subjects to the intervention, ensuring relevance to the clinical context of Alzheimer’s disease
2. Intervention: lecanemab	Lecanemab is the specific intervention of interest, allowing for a targeted analysis of its efficacy and safety in the context of Alzheimer’s disease	2. In vitro studies	In vitro studies are excluded to prioritize evidence derived from human subjects, contributing to the clinical applicability of the findings related to the intervention, lecanemab
3. Comparator: placebo	The inclusion of a placebo comparator enables a rigorous evaluation of the intervention’s effectiveness by providing a baseline for comparison	3. Observational or laboratory studies	Observational and laboratory studies are excluded to maintain the focus on interventional trials, ensuring the reliability of evidence regarding the efficacy and safety of lecanemab in patients with Alzheimer’s disease
4. Outcomes: efficacy and safety measures	The specified outcomes ensure a comprehensive assessment of both the effectiveness and safety of lecanemab in the treatment of Alzheimer’s disease	4. Overlapped results	Studies with overlapping results are excluded to avoid duplication and ensure that each included study contributes unique and valuable information to the overall synthesis
5. Study design: peer-reviewed randomized control trials (RCTs)	RCTs are considered the gold standard for evaluating intervention efficacy. Including only peer reviewed RCTs enhances the methodological rigor and reliability of the evidence	5. Reviews, books, posters, thesis, editorials, notes, letters, case reports, case series, and conferences	These publication types are excluded to maintain the focus on primary research with a higher level of evidence, ensuring the reliability and validity of the synthesized findings
6. No restriction on age, sex, place, ethnicity, and language	Removing demographic restrictions ensures a broader representation of the population, enhancing the generalizability of the findings to diverse groups of individuals with Alzheimer’s disease		

### Data extraction

The data extraction was conducted by two investigators (K.A. and A.A.A.) and cross-checked by K.A. and B.A. The extracted information encompasses study characteristics, participant details, intervention specifics, outcome measures, and funding sources. For quantitative data, (mean differences [standard error (SE)]) used for ADCOMS, ADAS-Cog14, and CDR-SB. In addition, we extract data for treatment-emergent adverse events (TEAE), amyloid-related imaging abnormalities hemorrhage (ARIA-H), and amyloid-related imaging abnormalities edema (ARIA-E) to evaluate the safety of lecanemab 10 mg/kg biweekly.

Regarding missing data, we made efforts to obtain it and calculate mean differences [SE] from the original studies. If this is not feasible, imputation techniques such as mean imputation, utilized to estimate missing values, recognize the potential introduction of bias into the analysis.

### Quality assessment

The quality of the studies was appraised independently by K.A. and A.A.A. and cross-checked by B.A. and H.A. We assessed the risk of bias in included studies using the Cochrane risk of bias tool for RCTs. This tool is transparent, reproducible, and facilitates evidence-based decision-making by identifying potential biases in study design and reporting [14].

### Data synthesis

Statistical analyses were conducted using R software 4.2.2. For binary variables, risk ratios (RR) with 95% confidence intervals (CI) were calculated. Continuous outcomes were analyzed using the mean differences [SE] with a 95% CI. Heterogeneity among studies was assessed using the Cochrane *Q p*-value and *I*^2^ statistic, with a random effects model and common effect model. Publication bias was evaluated using funnel plots and Egger’s regression test.

We would like to highlight that this systematic review and meta-analysis has been diligently registered on Prospero, an international prospective register of systematic reviews. The registration number for this study is CRD42023430184.

## Results

### Study selection and characteristics

The literature search yielded 380 records, of which 55 were duplicates. The remaining 325 articles were then evaluated based on title and abstract, with 292 deemed irrelevant and removed. The remaining 33 articles were evaluated using full text for eligibility, and 29 were excluded. The overall count of incorporated studies is 4.

**Fig. 1:**
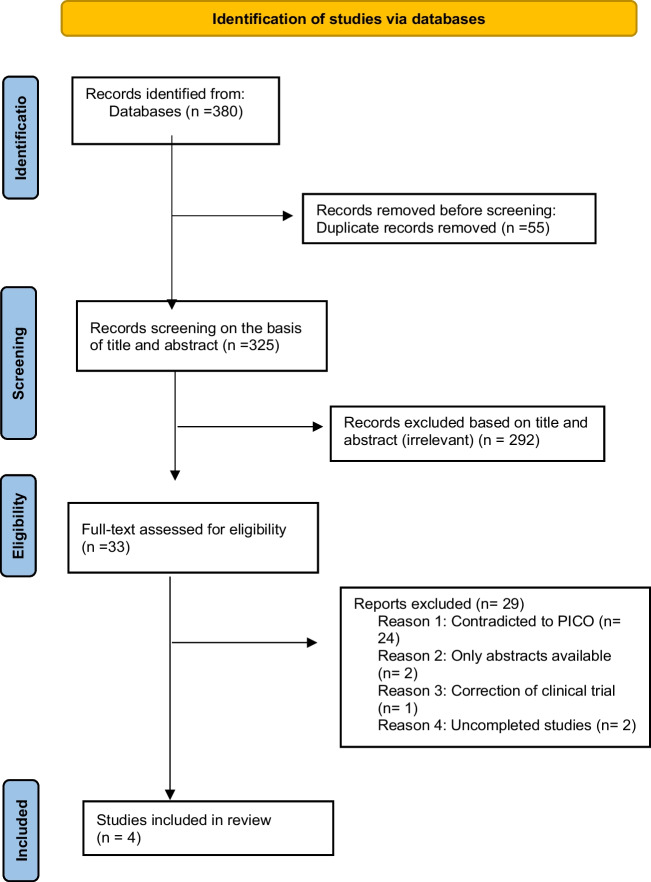
PRISMA flow chart of the studies’ identification and inclusion processes

Figure 1 outlines the schematic flow of the studies’ identification and inclusion processes. The characteristics of the studies included are shown in Table 2. Four articles comprising 2176 patients who received the treatment, and 1407 controls were included in this study.

**Table 2:** The characteristics of the included studies

Studies	Publisher journal	Registration	Year of publication	Country of patients	Study design	Study period	Number of participants (cases/controls)	Mean age of cases (SD)	Mean age of control (SD)	Gender of cases (female/male (%))	Gender of control (female/male (%))	Intervention	Follow-up period	Funding of the trial
Logovinsky et al. [13]	*Alzheimer’s Research & Therapy*	NCT01230853	2016	USA	A multicenter double-blind randomized placebo-controlled study was performed in subjects with mild to moderate AD	2010/2012	60/20	70.3 ± 9.81	70.0 ± 11.7	33.3/66.7%	25/75%	Lecanemab 10 mg/kg biweekly	264 days	Eisai Inc
McDade et al. [6]	*Alzheimer’s Research & Therapy*	NCT01767311	2022	North America, Western Europe, and Asia Pacific	Multinational, multicenter, double-blind, placebo-controlled, parallel-group study (core) employing Bayesian design with response-adaptive randomization with an OLE	_	609/245	74 ± 7.7	71.8 ± 8.2	48.3/51.7%	50/50%	Lecanemab 10 mg/kg biweekly	24 months	Eisai Inc
Swanson et al. [4]	*Alzheimer’s Research & Therapy*	NCT01767311	2021	North America, Europe, and Asia–Pacific	Multicenter, double-blind, placebo-controlled Bayesian design clinical trial	2012/2017	609/245	72.6 ± 8.8	71.1 ± 8.9	42/58%	58/42%	Lecanemab 10 mg/kg biweekly	18 months	Eisai Inc
Van Dyck et al. [12]	*The New England Journal of Medicine*	NCT03887455	2023	North America, Europe, and Asia	Multicenter, double-blind, placebo-controlled, parallel-group trial involving persons with early Alzheimer’s disease	2019/2021	898/897	71.4 ± 7.9	71.0 ± 7.8	51.6/48.4%	53.0/47.0%	Lecanemab 10 mg/kg biweekly	18 months	Eisai Inc and Biogen

McDade et al.’s study is an extension of the work presented in Swanson et al. Both studies are intricately connected, with McDade et al. building upon the findings and insights reported in Swanson et al. The term “extension” in this context indicates a sequential or follow-up study that delves deeper into specific aspects or expands upon the initial investigation presented in the earlier work.

The funding landscape for the discussed studies on lecanemab highlights the substantial support provided by pharmaceutical entities for advancing Alzheimer’s research and therapeutic development. Eisai Inc., a prominent pharmaceutical company, emerges as a key contributor, backing the trials conducted by Logovinsky et al., McDade et al., Swanson et al., and Van Dyck et al. These multinational, multicenter studies, published in esteemed journals such as *Alzheimer’s Research & Therapy* and *The New England Journal of Medicine*, collectively involved thousands of participants across various regions. The financial backing from Eisai Inc. demonstrates a strategic commitment to advancing the understanding and treatment of Alzheimer’s disease. The collaboration between academia and industry, evident in these studies, underscores the collaborative effort required to address the complex challenges posed by neurodegenerative diseases, with funding playing a pivotal role in driving such groundbreaking research endeavors.

## Interpreting gender differences in included studies

In comparing gender ratios between case and control groups across four studies, a notable variation emerges in the observed associations. Swanson et al.’s study reveals a significant disparity, with individuals in the study group having substantially lower odds of being in the case group compared to controls (*p* < 0.0001). Conversely, Van Dyck et al., McDade et al., and Logovinsky et al. report less pronounced differences, with *p*-values of 0.179, 0.688, and 0.488, respectively, failing to reach statistical significance (Table 3).

**Table 3:** Interpreting gender differences in included studies

Study	Gender		
	OR	95% CI	*p*-value
Swanson et al	0.526	0.390–0.710	< 0.0001
Van Dyck et al	1.135	0.944–1.366	0.179
McDade et al	0.940	0.699–1.266	0.688
Logovinsky et al	1.5	0.477–4.717	0.488

### Treatment effect

#### Lecanemab 10 mg/kg vs. placebo effect on ADCOMS change

The findings from our meta-analysis revealed a statistically significant decrease (*p*-value < 0.0001) in ADCOMS scores when lecanemab is administered at a 10 mg/kg dosage compared to a placebo. Both the common effect model and the random effects model estimate a mean difference of − 0.0508, and the narrow 95% confidence intervals suggest a consistent effect size across the studies.

**Fig. 2:**
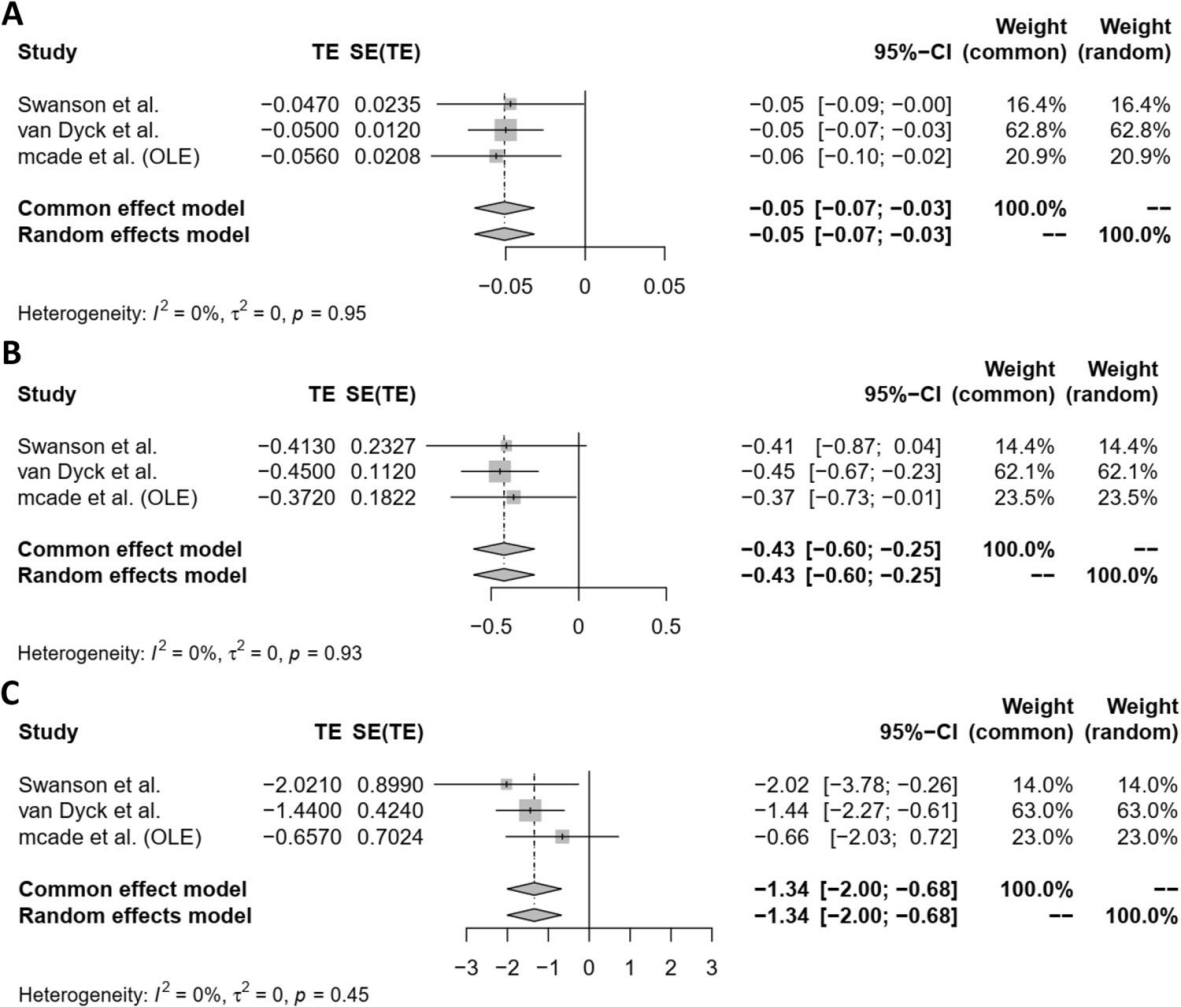
**A–C **Forest plots depicting lecanemab 10 mg/kg efficacy against AD parameters (Fig **A**: ADCOMS; Fig 2: CDR-SB; Fig 3: ADAS-cog14)

Furthermore, the absence of significant heterogeneity (*I*^2^ = 0.0%) among the studies implies a consensus regarding the impact of lecanemab on ADCOMS scores. The test of heterogeneity (*Q* statistic) yields a *p*-value of 0.9545, indicating a lack of significant heterogeneity, and suggesting a high degree of consistency in the findings across the studies (Fig. 2A). For further details, please refer to Sect. 1 in the supplementary file.

#### Lecanemab 10 mg/kg vs. placebo effect on CDR-SB change

The results of the meta-analysis suggest that the administration of lecanemab at a dosage of 10 mg/kg biweekly is linked to a statistically significant reduction (*p* < 0.0001) in CDR-SB scores compared to a placebo. Both the common effect model and the random effects model estimate a mean difference of approximately − 0.4264, with narrow 95% confidence intervals, indicating consistency in the effect size across the studies.

The absence of significant heterogeneity (*I*^2^ = 0.0%) among the studies indicates a consensus in their findings, further supporting the strength of the results. The test of heterogeneity (*Q* statistic) yields a *p*-value of 0.9339, signifying a lack of significant heterogeneity among the studies and suggesting relative consistency in their findings (Fig. 2B). For further details, please refer to Sect. 2 in the supplementary file.

#### Lecanemab 10 mg/kg vs. placebo effect on ADAS-cog14 change

The results of the meta-analysis indicate that administering lecanemab at a 10 mg/kg dosage biweekly leads to a statistically significant reduction (*p* < 0.0001) in ADAS-cog14 scores compared to a placebo in patients diagnosed with Alzheimer’s disease. Both the common effect model and the random effects model estimate a mean difference of approximately − 1.3416, with narrow 95% confidence intervals, highlighting a consistent effect size across studies.

The absence of significant heterogeneity (*I*^2^ = 0.0%) among the studies suggests agreement in their findings, further supporting the reliability of the results. The heterogeneity test (*Q* statistic) yields a *p*-value of 0.4550, indicating a lack of significant heterogeneity among the studies and suggesting a relatively consistent pattern in their findings (Fig. 2C). For further details, please refer to Sect. 3 in the supplementary file.

### Safety concerns

#### Lecanemab 10 mg/kg vs. placebo effect on any TEAE outcome

The meta-analysis results concerning the occurrence of TEAE when comparing lecanemab at a dosage of 10 mg/kg administered biweekly to placebo shows that the random effects model estimates a pooled relative risk (RR) of 0.6647. However, the 95% CI is wide indicating no effect, and the *p*-value is 0.3034, signifying a lack of statistical significance.

**Fig. 3:**
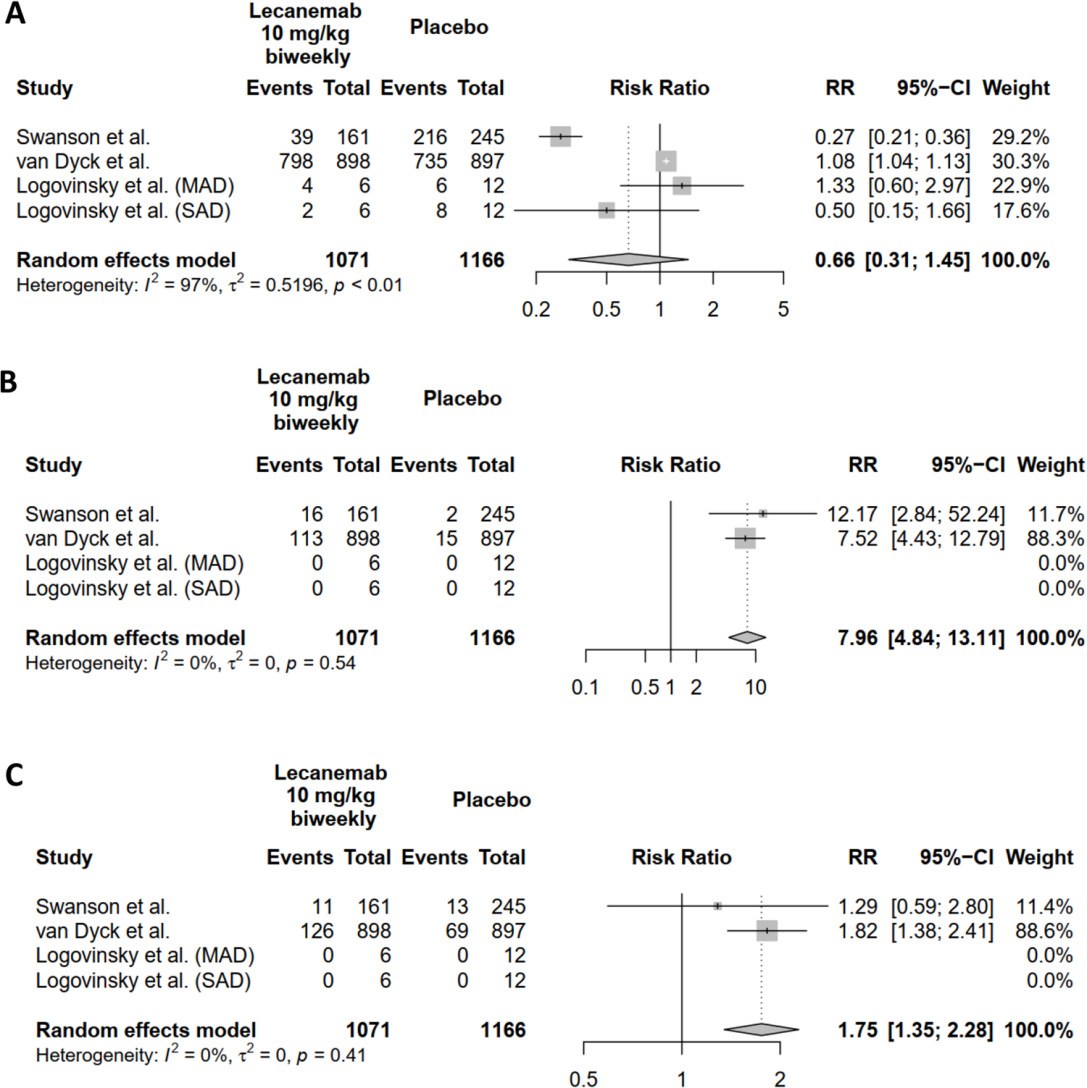
**A–C **The safety assessment of lecanemab at a dosage of 10 mg/kg in AD patients (Fig **A**: TEAE; Fig **B**: ARIA-E; Fig **C**: ARIA-H)

Additionally, there is significant heterogeneity among the studies, with an *I*^2^ of 96.8%. This suggests diverse findings regarding TEAE outcomes across the studies (Fig. 3A). For further details, please refer to Sect. 4 in the supplementary file.

#### Lecanemab 10 mg/kg vs. placebo effect on ARIA-E outcome

The meta-analysis findings for the occurrence of ARIA-E when comparing lecanemab at a dosage of 10 mg/kg administered biweekly to placebo are outlined as shows that the random effects model estimates a pooled RR of 7.9613, with a 95% CI of [4.8358; 13.1068], and a very low *p*-value (< 0.0001), indicating a statistically significant increased risk of ARIA-E associated with lecanemab treatment.

Furthermore, there is no significant heterogeneity among the studies, as indicated by the *p*-value of 0.5430 and an *I*^2^ of 0.0%. This implies that the studies are consistent in their findings regarding the risk of ARIA-E (Fig. 3B). For further details, please refer to Sect. 5 in the supplementary file.

#### Lecanemab 10 mg/kg vs. placebo effect on ARIA-H outcome

The meta-analysis results for the occurrence of ARIA-H when comparing lecanemab at a dosage of 10 mg/kg administered biweekly to placebo reveal that the pooled RR estimated by the random effects model is 1.7533, with a 95% CI of [1.3488; 2.2790], and a very low *p*-value (< 0.0001). This indicates a statistically significant increased risk of ARIA-H associated with lecanemab treatment.

Moreover, there is no significant heterogeneity among the studies, as indicated by the *p*-value of 0.4088 and an *I*^2^ of 0.0%. This suggests that the studies are consistent in their findings regarding the risk of ARIA-H (Fig. 3C). Retrieved data related to adverse events are depicted in Table 4. For further details, please refer to Sect. 6 in the supplementary file.

**Table 4:** List for adverse events

Studies	Any TEAE		ARIA-E		ARIA-H	
	No. of patients		No. of patients		No. of patients	
	Placebo (*n* (%))	Lecanemab 10 mg/kg biweekly (n (%))	Placebo (*n* (%))	Lecanemab 10 mg/kg biweekly (*n* (%))	Placebo (*n* (%))	Lecanemab 10 mg/kg biweekly (*n* (%))
Logovinsky et al. (MAD)	6 (50)	4 (66.67)	0 (0)	0 (0)	0 (0)	0 (0)
Logovinsky et al. (SAD)	8 (66.67)	2 (33.33)	0 (0)	0 (0)	0 (0)	0 (0)
Swanson et al	216 (88.16)	39 (24.22)	2 (0.82)	16 (9.94)	13 (5.31)	11 (6.83)
Van Dyck et al	735 (81.94)	798 (88.86)	15 (1.67)	113 (12.58)	69 (7.69)	126 (14.03)

### Publication bias testing and sensitivity analyses

**Fig. 4:**
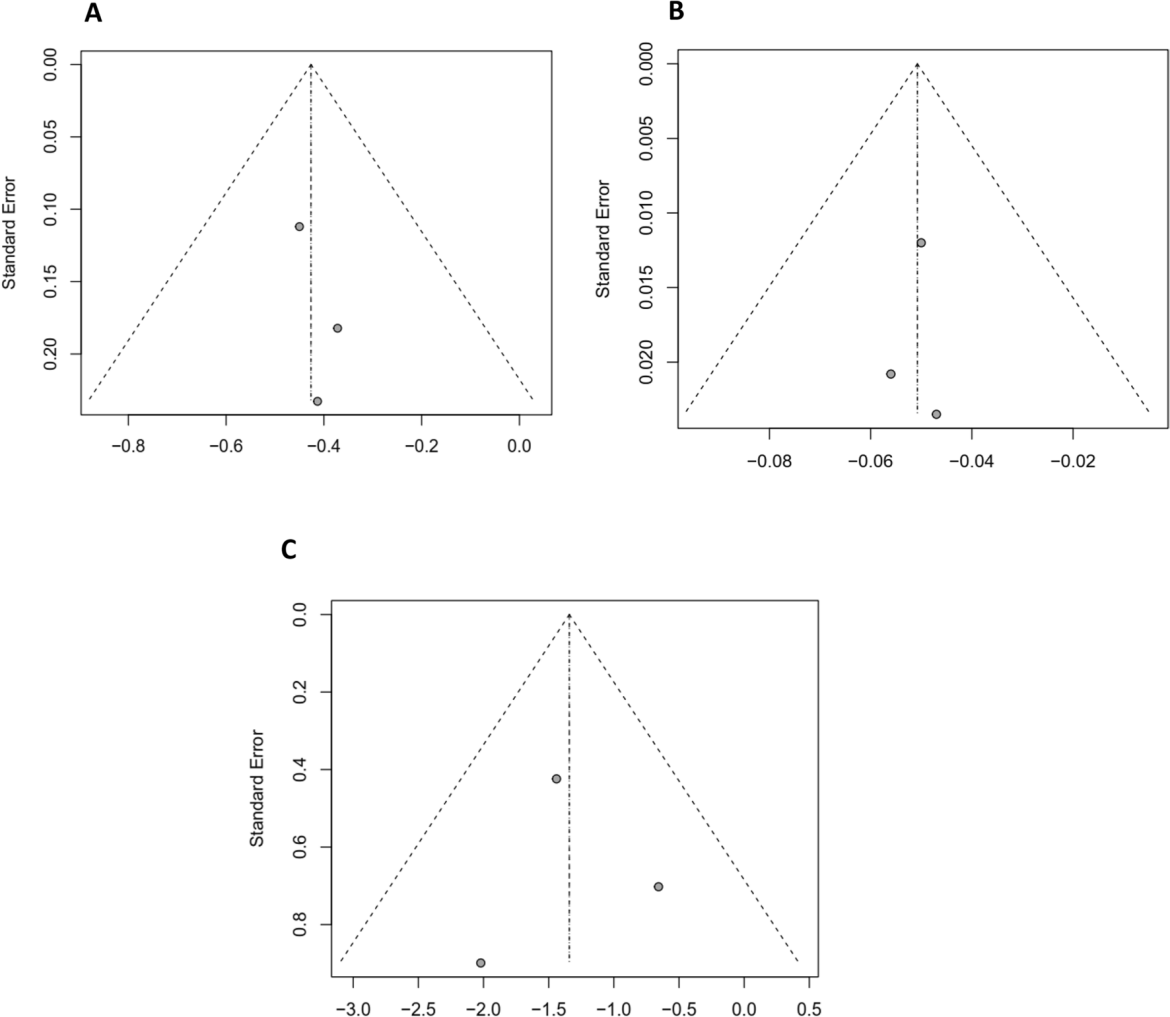
**A–C** Funnel plots and Egger’s tests evaluating the publication bias (Fig **A**: ADCOMS; Fig 2: CDR-SB; Fig 3: ADAS-cog14)

Funnel plots and Egger’s tests were carried out to evaluate the publication bias (Fig. 4A, B, C). The results indicated no evidence of significant publication bias, as demonstrated by the Egger’s test with a *p*-value greater than 0.05.

### The results of the regression test for funnel plot asymmetry for ADCOMS

The results of the regression test for funnel plot asymmetry suggest that there is no statistically significant evidence of publication bias or funnel plot asymmetry in the meta-analysis. The *p*-value of 0.9564 indicates that the relationship between study precision SE and effect size is not significantly different from what would be expected by chance. In other words, the distribution of studies in the funnel plot appears to be symmetric and not skewed due to publication bias.

The limit estimate as SE approaches zero gives an estimate of the effect size under ideal conditions, if the studies had perfect precision. The point estimate of − 0.0491 suggests that, under these ideal conditions, the mean difference or effect size is approximately − 0.0491. However, the wide confidence interval (− 0.1117 to 0.0136) indicates substantial uncertainty in this estimate.

### The results of the regression test for funnel plot asymmetry for CDR-SB

The results of the regression test for funnel plot asymmetry suggest that there is no statistically significant evidence of publication bias or funnel plot asymmetry in the meta-analysis. The *p*-value of 0.7808 indicates that the relationship between study precision SE and effect size is not significantly different from what would be expected by chance. In other words, the distribution of studies in the funnel plot appears to be symmetric and not skewed due to publication bias.

The limit estimate as SE approaches zero gives an estimate of the effect size under ideal conditions, assuming that the studies had perfect precision. The point estimate of − 0.5044 suggests that, under these ideal conditions, the mean difference or effect size is approximately − 0.5044. However, the wide confidence interval (− 1.0807 to 0.0719) indicates substantial uncertainty in this estimate.

### The results of the regression test for funnel plot asymmetry for ADAS-cog14

The results of the regression test for funnel plot asymmetry suggest that there is no statistically significant evidence of publication bias or funnel plot asymmetry in the meta-analysis. The *p*-value of 0.8812 indicates that the relationship between study precision SE and effect size is not significantly different from what would be expected by chance. In other words, the distribution of studies in the funnel plot appears to be symmetric and not skewed due to publication bias.

The limit estimate as SE approaches zero gives an estimate of the effect size under ideal conditions, assuming that the studies had perfect precision. The point estimate of − 1.1020 suggests that, under these ideal conditions, the mean difference or effect size is approximately − 1.1020. However, the wide confidence interval (− 4.2541 to 2.0502) indicates substantial uncertainty in this estimate.

### Variability and heterogeneity

The heterogeneity *p*-value is a statistical measure that helps assess whether there is significant variability in the effect sizes observed across different studies included in a meta-analysis. A low *p*-value (typically less than 0.05) suggests the presence of significant heterogeneity, indicating that the variation in effect sizes is not likely due to chance alone.

The Egger’s test *p*-value > 0.05 generally indicates no evidence of significant publication bias. The *I*^2^ value represents the proportion of total variability across studies due to heterogeneity.

These results suggest a lack of significant heterogeneity in the analyses related to ADCOMS Change, CDR-SB Change, and ADAS-cog14 Change, with *I*^2^ values of 0.0%. The Egger’s test for publication bias yielded non-significant results across all analyses, indicating a relatively consistent pattern in the findings without substantial publication bias. However, the high heterogeneity (*I*^2^ = 96.8%) and statistically significant *p*-value (< 0.0001) for the “Any TEAE Outcome” indicate substantial variability in the TEAE outcomes across the included studies this variability may be due to the huge difference in sample size between studies. This level of heterogeneity suggests that there might be differences in study populations, interventions, or methodologies that contribute to the observed variation in TEAE outcomes (Table 5).

**Table 5:** Variability and heterogeneity

Title	Heterogeneity *p*-value	*I*^2^ value	Egger’s test *p*-value
ADCOMS Change	0.9545	0.0%	> 0.05
CDR-SB Change	0.9339	0.0%	> 0.05
ADAS-cog14 Change	0.4550	0.0%	> 0.05
Any TEAE Outcome	< 0.0001	96.8%	0.9998
ARIA-E Outcome	0.5430	0.0%	> 0.05
ARIA-H Outcome	0.4088	0.0%	> 0.05

### Quality assessment

**Fig. 5:**
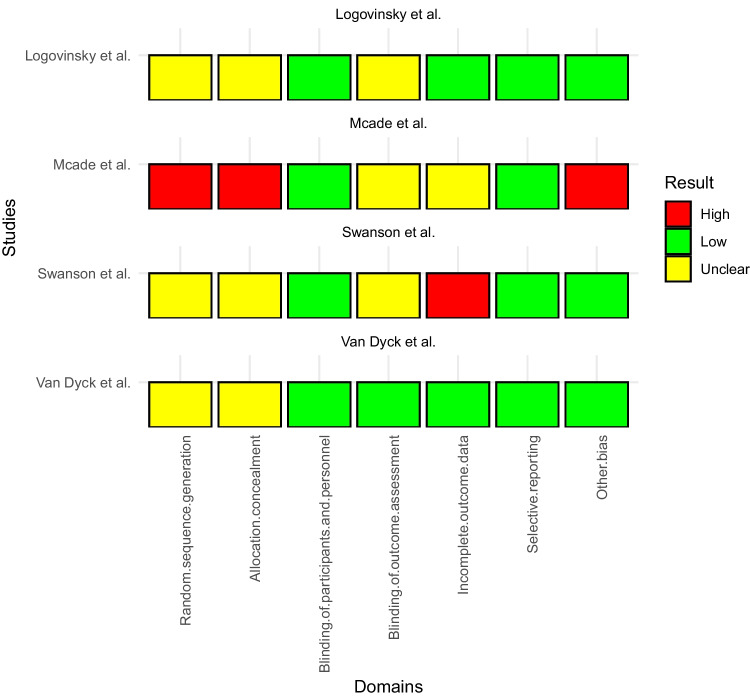
Variations in the methodological rigor and potential bias in included studies via the Cochrane risk of bias assessment tool

The quality of research design and reporting was assessed in several studies by Swanson et al., Logovinsky et al., Van Dyck et al., and Mcade et al. The analyzed studies present varying levels of bias across different domains. Swanson et al. and Logovinsky et al. have low risk of bias in multiple domains, particularly in terms of selection and performance. Van Dyck et al. demonstrate consistently low risk across all domains. On the other hand, Mcade et al. exhibit high risk in the selection and performance domains. Overall, domains such as selection and performance show lower bias across the studies, while attention to minimizing bias is warranted in domains such as detection and attrition, particularly in the study by Mcade et al. These findings indicate variations in the methodological rigor and potential bias in these studies (Fig. 5) (Table 6). For further details, please refer to Sect. 7 in the supplementary file.

**Table 6:** Cochrane risk of bias assessment tool for RCTs

Study	Random sequence generation	Allocation concealment	Blinding of participants and personnel	Blinding of outcome assessment	Incomplete outcome data	Selective reporting	Other bias
Logovinsky et al	Unclear	Unclear	Low	Unclear	Low	Low	Low
Mcade et al	High	High	Low	Unclear	Unclear	Low	High
Swanson et al	Unclear	Unclear	Low	Unclear	High	Low	Low
Van Dyck et al	Unclear	Unclear	Low	Low	Low	Low	Low

## Discussion

### Our efficacy results in perspective to other literature

The examination of lecanemab at a dosage of 10 mg/kg versus a placebo reveals a substantial and statistically significant positive impact on ADCOMS, CDR-SB, and ADAS-cog14 scores in patients with Alzheimer’s disease (AD) (*p* < 0.0001). This discovery implies that administering lecanemab at this specific dose elicits a beneficial effect in terms of slowing the progression of the disease.

The validity of these results is further bolstered by the consistency observed across the studies. The narrow 95% confidence intervals, common effect model, and random effects model all estimate very similar mean differences. The absence of significant heterogeneity among the studies (*p* > 0.05) underscores the consensus in their findings.

The evidence from this meta-analysis strongly supports the idea that lecanemab at a dosage of 10 mg/kg, administered biweekly, leads to a significant reduction in the evaluated cognitive and functional scores in AD patients. These findings hold promise for the potential efficacy of lecanemab as a treatment option in this context.

The FDA granted accelerated approval to lecanemab (Leqembi) for Alzheimer’s disease, despite uncertainties about its impact on patients. This approval was based on phase 2 data indicating a reduction in amyloid β plaques in early-stage patients. Lecanemab, priced at $26,500 per patient per year, targets and removes Aβ aggregates associated with the disease [15]. While the phase 2 trial did not meet its 12-month primary endpoint, it showed reduced brain amyloid and consistent clinical improvement at 18 months [6]. Phase 3 results in the *New England Journal of Medicine* revealed reduced amyloid markers and moderately less cognitive and functional decline after 18 months with lecanemab compared to a placebo [12]. This accelerated approval allows the drug to be marketed before full effectiveness is proven.

A separate meta-analysis reinforces our results, demonstrating that lecanemab brings about statistically significant slower decline in cognition, functionality, and behavior among patients in the early stages of Alzheimer’s disease [16]. However, it is important to note that the methodology of this analysis is vague and still requires further investigation and clarification.

A systematic review and meta-analysis of randomized clinical trials conducted by Yue Qiao et al. found that lecanemab was beneficial to stabilize or slow down the decrease in CDR-SB, ADCOMS, and ADAS-cog in patients with early AD. The study also reported that lecanemab showed significant positive statistical efficacy with respect to cognition, function, and behavior in patients with early AD though the actual clinical significance is yet to be established [17].

One phase 3 trial has been initiated with the objective of validating the effectiveness and safety of lecanemab, the AHEAD trial (NCT04468659) for preclinical Alzheimer’s disease. The 18-month data from the phase 2 Study 201 were instrumental in properly powering the Clarity AD trial, which recently published positive preliminary results for a variety of robust outcomes [18].

While lecanemab did show a statistically significant reduction in the rate of cognitive decline, it may not necessarily translate to clinically meaningful changes, as indicated by published data on the Minimal Clinically Important Difference (MCID) and the opinions of certain clinical experts [19].

Lecanemab treatment was estimated to slow disease progression, extending the duration of mild cognitive impairment due to Alzheimer’s disease (MCI due to AD) and mild AD dementia while shortening the time to reach moderate and severe AD dementia. On average, it added 2.51, 3.13, and 2.34 years to the time to reach mild, moderate, and severe AD dementia, respectively. However, the model faces limitations: Mortality risk and patient utility data sources are uncertain. It solely focused on cognition’s indirect impact via CDR-SB, neglecting other severity domains and composite measures. Simulated population profiles might not accurately reflect those of Study 201. Severity projection leaned heavily on CDR-SB alone, with simplistic estimates for institutional care risk and patient utility [20].

### Our safety results in perspective to other literature

Lecanemab at 10 mg/kg, administered biweekly, was assessed for its safety on three outcomes: The meta-analysis found no statistically significant difference in TEAE occurrence between lecanemab and placebo. The results were not conclusive and showed substantial variation among the studies. The analysis revealed a significant, increased risk of ARIA-E associated with lecanemab treatment at 10 mg/kg, administered biweekly, compared to placebo. This risk was consistent across studies.

Also indicated a notable, statistically significant increased risk of ARIA-H linked to lecanemab treatment at the same dosage (10 mg/kg) and frequency compared to placebo. This risk was consistent among the included studies and raises concerns about the safety of lecanemab, particularly regarding ARIA-H.

In certain studies, a risk ratio of 0 in the treatment group compared to the control group suggests no adverse events among treated patients. However, caution is needed due to the small sample size. Further research with larger cohorts is necessary to validate these findings and understand the clinical significance of the observed protective effect, which refers to a reduction in the risk or severity of adverse outcomes associated with a particular intervention or factor.

In both the phase 2 core and OLE studies, the occurrence of ARIA-E was infrequent, affecting less than 10% of participants, and symptomatic cases were below 3%. ARIA-E is typically presented as asymptomatic, mild to moderately severe, and manifested within the initial three months of treatment. The incidence of ARIA-E was associated with the highest concentration of lecanemab in the bloodstream and was more common in individuals who were homozygous carriers of apolipoprotein E4 (ApoE4). Notably, ARIA-H and ARIA-E had a similar occurrence rate in both the core and OLE phases of the study [21].

Subgroup analyses revealed that lecanemab had a more pronounced effect in slowing cognitive decline in individuals who are ApoE ε4 noncarriers compared to carriers and had the least impact on participants who were ApoE ε4 homozygotes. Since ApoE ε4 carriers are at a higher risk of experiencing ARIA, it is important to carefully consider the balance between benefits and potential harms for such patients [22].

In the base-case analysis, it resulted in an increase of 0.73 life years (LY) and 0.75 quality-adjusted LYs (QALY), with a reduction of 0.03 years in caregiver QALYs lost. The model also predicted a lower lifetime likelihood of institutional care admission for those receiving lecanemab alongside standard care (25% versus 31%) [20].

### Results from the Cochrane risk of bias assessment

Evaluating research by Swanson, Logovinsky, Van Dyck, and Mcade uncovers differing levels of bias. Swanson and Logovinsky show minimal bias, particularly in selection and performance. Van Dyck consistently maintains low bias. In contrast, Mcade raises concerns with higher bias, particularly in selection and performance domains. In separate meta-analyses, they used the cochrane risk of bias tool. However, their results slightly differ from ours; they found unclear risk of bias in performance and detection, in contrast to our findings [16].

### Future research recommendations

Suggest initiating comprehensive, long-term safety studies to enhance insights. Conduct comparative safety analyses, directly comparing lecanemab with established Alzheimer’s treatments like donepezil or rivastigmine. Collaboratively pinpoint specific risk factors and incorporate patient-reported outcomes for a thorough comprehension of lecanemab’s impact. Implement trials across diverse geographic locations and various ethnicities to ensure global efficacy and safety assessments.

### Strengths and limitations

In our meta-analysis, we specifically emphasized the efficacy and safety of lecanemab at a dosage of 10 mg/kg—the same dose available on the market. Our focus was to address any inconsistencies in the findings among various trials, ensuring clarity regarding the drug’s performance. By concentrating on this specific dosage, we aimed to provide a comprehensive understanding of lecanemab’s safety and efficacy for consumers, thereby resolving potential conflicts in its profile across different studies.

We were compelled to compute the mean difference and standard error (SE) for the meta-analysis since the studies did not support the use of standard deviation (SD). We reached out to Eisai Company to request this information, but they responded that they would discuss the matter and provide a decision. Unfortunately, we did not receive any further communication from them. In the case of the study by Mcade et al., we had to estimate the SE by imputing data from other studies due to insufficient information for SE calculation.

There are inconsistencies between the Prospero registration and the full text. In the registration, we stated that studies in languages other than English would be excluded, but we discovered four studies without English translations, all of which were either irrelevant or mere reviews. Additionally, we planned to assess the mini-mental state examination (MMSE) and positron emission tomography standardized uptake value ratio (PET SUVr), but the available data proved insufficient.

## Conclusion

The lack of notable heterogeneity, except for a significant heterogeneity observed in the case of TEAE with an *I*^2^ of 96.8%, underscores the reliability of the efficacy results. The consistent outcomes across the studies affirm the dependability of the findings. The meta-analysis indicates that administering lecanemab at a dosage of 10 mg/kg biweekly is linked to a positive impact on enhancing cognitive outcomes measured across various parameters in individuals with AD. These results provide valuable insights into the potential effectiveness of lecanemab as a therapeutic intervention for addressing cognitive impairment in this patient population.

While no strong evidence supports a significant difference in the occurrence of TEAE, the meta-analysis raises substantial safety concerns related to the increased risk of ARIA-E and ARIA-H associated with lecanemab at a dosage of 10 mg/kg administered biweekly when compared to placebo. These findings, particularly the elevated risk of ARIA-H, highlight the need for careful consideration and monitoring of the safety profile of lecanemab in clinical practice.

### Supplementary Information

Below is the link to the electronic supplementary material.

Supplementary file1 (DOCX 25 KB)

## Data Availability

All data generated or analyzed during this study are included in this published article (and its supplementary information file).
