# Supplementary material for: The efficacy and safety of lecanemab 10 mg/kg biweekly compared to a placebo in patients with Alzheimer’s disease: a systematic review and meta-analysis of randomized controlled trials
Source: Neurol Sci. 2024 Apr 3;45(8):3583–97. doi: 10.1007/s10072-024-07477-w (PMC11254984; doi:10.1007/s10072-024-07477-w)
Supplement: Supplementary file 1 — Supplementary file1 (DOCX 25 KB) [file 10072_2024_7477_MOESM1_ESM.docx]

**Section 1**

ADCOMS

| **Studies** | **Mean Difference** | **SE** |
| --- | --- | --- |
| Swanson et al. | -0.047 | 0.0235 |
| van Dyck et al. | -0.05 | 0.012 |
| mcade et al. (OLE) | -0.056 | 0.0208 |

Study-Level Results:

Swanson et al.: The mean difference is -0.047, with a 95% confidence interval (CI) of [-0.0931; -0.0009]. The weight assigned to this study in the common effect model is 16.4% and in the random effects model is also 16.4%.

van Dyck et al.: The mean difference is -0.050, with a 95% CI of [-0.0735; -0.0265]. This study has the highest weight in both the common and random effects models at 62.8%.

mcade et al. (OLE): The mean difference is -0.056, with a 95% CI of [-0.0968; -0.0152]. This study has a weight of 20.9% in both models.

Pooled Effect Size:

Common effect model: The pooled mean difference is -0.0508 with a 95% CI of [-0.0694; -0.0321]. This model assumes that there is a single true effect size common to all studies.

Random effects model: The pooled mean difference is also -0.0508 with the same 95% CI. The random effects model considers both within-study and between-study variability.

Heterogeneity:

The test of heterogeneity (Q statistic) shows a p-value of 0.9545, indicating that there is no significant heterogeneity among the studies. This suggests that the studies are relatively consistent in their findings.

The I² statistic, which quantifies the percentage of total variation across studies that is due to heterogeneity, is 0.0%. This further supports the low level of heterogeneity.

Conclusion:

The meta-analysis results indicate that, on average, Lecanemab at a dosage of 10mg/kg is associated with a statistically significant decrease in ADCOMS scores compared to placebo. The common effect model and the random effects model both estimate a mean difference of -0.0508 with narrow 95% CIs. This suggests that the effect size is consistent across the studies, and the results are robust.

Additionally, the lack of significant heterogeneity (I² = 0.0%) suggests that the studies are in agreement regarding the effect of Lecanemab on ADCOMS scores.

In summary, based on this meta-analysis, there is evidence to support that Lecanemab at a dosage of 10mg/kg has a beneficial effect in reducing ADCOMS scores in Alzheimer's disease patients compared to placebo. However, while the effect is statistically significant, the clinical significance should also be considered, and further research and evaluation are needed to fully assess the implications of this treatment in real-world clinical practice.

**Section 2**

CDR-SB

| **Studies** | **Mean Difference** | **SE** |
| --- | --- | --- |
| Swanson et al. | -0.413 | 0.2327 |
| van Dyck et al. | -0.45 | 0.112 |
| mcade et al. (OLE) | -0.372 | 0.1822 |

Study-Level Results:

Swanson et al.:

Mean Difference: -0.413

95% Confidence Interval (CI): [-0.8691; 0.0431]

Weight in Common Effect Model: 14.4%

Weight in Random Effects Model: 14.4%

van Dyck et al.:

Mean Difference: -0.45

95% Confidence Interval (CI): [-0.6695; -0.2305]

Weight in Common Effect Model: 62.1%

Weight in Random Effects Model: 62.1%

mcade et al. (OLE):

Mean Difference: -0.372

95% Confidence Interval (CI): [-0.7291; -0.0149]

Weight in Common Effect Model: 23.5%

Weight in Random Effects Model: 23.5%

Pooled Effect Size:

Common effect model:

Pooled Mean Difference: -0.4264

95% CI: [-0.5994; -0.2533]

p-value: < 0.0001

Random effects model:

Pooled Mean Difference: -0.4264

95% CI: [-0.5994; -0.2533]

p-value: < 0.0001

Heterogeneity:

The test of heterogeneity (Q statistic) shows a p-value of 0.9339, indicating that there is no significant heterogeneity among the studies. This suggests that the studies are relatively consistent in their findings.

The I² statistic is 0.0%, which indicates that no significant heterogeneity exists among the studies. This further supports the consistency of the results.

Conclusion:

The meta-analysis results suggest that Lecanemab at a dosage of 10mg/kg administered biweekly is associated with a statistically significant reduction in CDR-SB scores compared to placebo.

The common effect model and the random effects model estimate a mean difference of approximately -0.4264, with narrow 95% confidence intervals.

The lack of significant heterogeneity (I² = 0.0%) indicates that the studies are consistent in their findings, supporting the robustness of the results.

Overall, based on this meta-analysis, there is evidence to suggest that Lecanemab at a dosage of 10mg/kg administered biweekly has a beneficial effect in reducing CDR-SB scores in patients. However, while the effect is statistically significant, the clinical significance and potential side effects should be considered. Further research and clinical evaluation are necessary to fully assess the implications of this treatment in the context of dementia and Alzheimer's disease.

The meta-analysis results suggest that Lecanemab at a dosage of 10mg/kg administered biweekly is associated with a statistically significant reduction in CDR-SB (Clinical Dementia Rating Sum of Boxes) scores compared to placebo.

The common effect model and the random effects model estimate a mean difference of approximately -0.4264, with narrow 95% confidence intervals.

The p-value, which is less than 0.0001, indicates the statistical significance of the effect.

The lack of significant heterogeneity (I² = 0.0%) indicates that the studies are consistent in their findings, supporting the robustness of the results.

Overall, based on this meta-analysis, there is strong evidence to suggest that Lecanemab at a dosage of 10mg/kg administered biweekly has a beneficial effect in reducing CDR-SB scores in patients. The statistical significance (p < 0.0001) reinforces the confidence in this conclusion. However, while the effect is statistically significant, the clinical significance and potential side effects should be considered. Further research and clinical evaluation are necessary to fully assess the implications of this treatment in the context of dementia and Alzheimer's disease.

**Section 3**

ADAS-cog14

| **Studies** | **Mean Difference** | **SE** |
| --- | --- | --- |
| Swanson et al. | -2.021 | 0.899 |
| van Dyck et al. | -1.44 | 0.424 |
| mcade et al. (OLE) | -0.657 | 0.7024 |

Study-Level Results:

Swanson et al.:

Mean Difference: -2.0210

95% Confidence Interval (CI): [-3.7830; -0.2590]

Weight in Common Effect Model: 14.0%

Weight in Random Effects Model: 14.0%

van Dyck et al.:

Mean Difference: -1.4400

95% Confidence Interval (CI): [-2.2710; -0.6090]

Weight in Common Effect Model: 63.0%

Weight in Random Effects Model: 63.0%

mcade et al. (OLE):

Mean Difference: -0.6570

95% Confidence Interval (CI): [-2.0337; 0.7197]

Weight in Common Effect Model: 23.0%

Weight in Random Effects Model: 23.0%

Pooled Effect Size:

Common effect model:

Pooled Mean Difference: -1.3416

95% CI: [-2.0013; -0.6819]

p-value: < 0.0001

Random effects model:

Pooled Mean Difference: -1.3416

95% CI: [-2.0013; -0.6819]

p-value: < 0.0001

Heterogeneity:

The test of heterogeneity (Q statistic) shows a p-value of 0.4550, indicating that there is no significant heterogeneity among the studies. This suggests that the studies are relatively consistent in their findings.

The I² statistic is 0.0%, which indicates that no significant heterogeneity exists among the studies. This further supports the consistency of the results.

Conclusion:

The meta-analysis results suggest that Lecanemab at a dosage of 10mg/kg administered biweekly is associated with a statistically significant reduction in ADAS-cog14 scores compared to placebo in patients with Alzheimer's disease.

The common effect model and the random effects model estimate a mean difference of approximately -1.3416, with narrow 95% confidence intervals.

The p-value, which is less than 0.0001, indicates the statistical significance of the effect.

The lack of significant heterogeneity (I² = 0.0%) indicates that the studies are consistent in their findings, supporting the robustness of the results.

Overall, based on this meta-analysis, there is strong evidence to suggest that Lecanemab at a dosage of 10mg/kg administered biweekly has a beneficial effect in reducing ADAS-cog14 scores in patients with Alzheimer's disease. The statistical significance (p < 0.0001) reinforces the confidence in this conclusion. However, while the effect is statistically significant, the clinical significance and potential side effects should be considered. Further research and clinical evaluation are necessary to fully assess the implications of this treatment in the context of Alzheimer's disease.

**Section 4**

Adverse events

Any TAEA

Study-Level Results:

Swanson et al.:

Relative Risk (RR): 0.2748

95% Confidence Interval (CI): [0.2083; 0.3625]

Weight in Random Effects Model: 29.2%

van Dyck et al.:

Relative Risk (RR): 1.0845

95% Confidence Interval (CI): [1.0436; 1.1270]

Weight in Random Effects Model: 30.3%

Logovinsky et al. (MAD):

Relative Risk (RR): 1.3333

95% Confidence Interval (CI): [0.5990; 2.9678]

Weight in Random Effects Model: 22.9%

Logovinsky et al. (SAD):

Relative Risk (RR): 0.5000

95% Confidence Interval (CI): [0.1506; 1.6604]

Weight in Random Effects Model: 17.6%

Pooled Effect Size:

Random effects model:

Pooled Relative Risk (RR): 0.6647

95% CI: [0.3054; 1.4467]

p-value: 0.3034

Heterogeneity:

The test of heterogeneity (Q statistic) shows a p-value of < 0.0001, indicating significant heterogeneity among the studies. This suggests that the studies have diverse findings regarding TEAE outcomes.

The I² statistic is 96.8%, which indicates a high level of heterogeneity. This suggests that the variability in TEAE outcomes among the studies is substantial.

Conclusion:

The meta-analysis results for the occurrence of treatment-emergent adverse events (TEAE) when comparing Lecanemab at a dosage of 10mg/kg administered biweekly to placebo are as follows:

The pooled Relative Risk (RR) estimated by the random effects model is 0.6647. However, the 95% CI is wide and includes 1 (no effect), and the p-value is 0.3034, indicating that the effect is not statistically significant.

There is significant heterogeneity among the studies, with an I² of 96.8%. This suggests that the studies have diverse findings regarding TEAE outcomes.

In summary, the meta-analysis does not provide strong evidence for a significant difference in the occurrence of TEAE between Lecanemab and placebo, as the result is not statistically significant. The high heterogeneity among the studies further complicates the interpretation. This suggests that TEAE outcomes may vary across different trials. Further research and investigation may be required to better understand the safety profile and potential risks associated with Lecanemab in clinical practice.

**Section 5**

ARIA-E

Study-Level Results:

Swanson et al.:

Relative Risk (RR): 12.1739

95% Confidence Interval (CI): [2.8372; 52.2352]

Weight in Random Effects Model: 11.7%

van Dyck et al.:

Relative Risk (RR): 7.5249

95% Confidence Interval (CI): [4.4266; 12.7920]

Weight in Random Effects Model: 88.3%

Logovinsky et al. (MAD):

Data Not Available (NA)

Weight in Random Effects Model: 0.0%

Logovinsky et al. (SAD):

Data Not Available (NA)

Weight in Random Effects Model: 0.0%

Pooled Effect Size:

Random effects model:

Pooled Relative Risk (RR): 7.9613

95% CI: [4.8358; 13.1068]

p-value: < 0.0001

Heterogeneity:

The test of heterogeneity (Q statistic) shows a p-value of 0.5430, indicating no significant heterogeneity among the studies. This suggests that the studies are relatively consistent in their findings regarding ARIA-E.

The I² statistic is 0.0%, which indicates no significant heterogeneity. This further supports the consistency of the results.

Conclusion:

The meta-analysis results for the occurrence of ARIA-E when comparing Lecanemab at a dosage of 10mg/kg administered biweekly to placebo are as follows:

The pooled Relative Risk (RR) estimated by the random effects model is 7.9613, with a 95% CI of [4.8358; 13.1068], and a very low p-value (< 0.0001). This indicates a statistically significant increased risk of ARIA-E associated with Lecanemab treatment.

There is no significant heterogeneity among the studies, as indicated by the p-value of 0.5430 and an I² of 0.0%. This suggests that the studies are consistent in their findings regarding the risk of ARIA-E.

In summary, the meta-analysis suggests that there is a statistically significant increased risk of ARIA-E associated with the administration of Lecanemab at a dosage of 10mg/kg administered biweekly when compared to placebo. The results are consistent across the included studies, and the risk is substantial, as indicated by the RR of 7.9613. This finding raises concerns about the safety profile of Lecanemab, and further research and clinical evaluation are needed to assess the implications of this risk in the context of Alzheimer's disease treatment. Patient safety and careful monitoring for ARIA-E should be a priority in clinical practice.

**Section 6**

ARIA-H

Study-Level Results:

Swanson et al.:

Relative Risk (RR): 1.2876

95% Confidence Interval (CI): [0.5915; 2.8031]

Weight in Random Effects Model: 11.4%

van Dyck et al.:

Relative Risk (RR): 1.8241

95% Confidence Interval (CI): [1.3806; 2.4100]

Weight in Random Effects Model: 88.6%

Logovinsky et al. (MAD):

Data Not Available (NA)

Weight in Random Effects Model: 0.0%

Logovinsky et al. (SAD):

Data Not Available (NA)

Weight in Random Effects Model: 0.0%

Pooled Effect Size:

Random effects model:

Pooled Relative Risk (RR): 1.7533

95% CI: [1.3488; 2.2790]

p-value: < 0.0001

Heterogeneity:

The test of heterogeneity (Q statistic) shows a p-value of 0.4088, indicating no significant heterogeneity among the studies. This suggests that the studies are relatively consistent in their findings regarding ARIA-H.

The I² statistic is 0.0%, which indicates no significant heterogeneity. This further supports the consistency of the results.

Conclusion:

The meta-analysis results for the occurrence of ARIA-H when comparing Lecanemab at a dosage of 10mg/kg administered biweekly to placebo are as follows:

The pooled Relative Risk (RR) estimated by the random effects model is 1.7533, with a 95% CI of [1.3488; 2.2790], and a very low p-value (< 0.0001). This indicates a statistically significant increased risk of ARIA-H associated with Lecanemab treatment.

There is no significant heterogeneity among the studies, as indicated by the p-value of 0.4088 and an I² of 0.0%. This suggests that the studies are consistent in their findings regarding the risk of ARIA-H.

In summary, the meta-analysis suggests that there is a statistically significant increased risk of ARIA-H associated with the administration of Lecanemab at a dosage of 10mg/kg administered biweekly when compared to placebo. The results are consistent across the included studies, and the risk is substantial, as indicated by the RR of 1.7533. This finding raises concerns about the safety profile of Lecanemab, particularly regarding the risk of ARIA-H. Further research and clinical evaluation are needed to assess the implications of this risk in the context of Alzheimer's disease treatment, and careful monitoring for ARIA-H is essential in clinical practice.

**Section 7**

Cochrane Risk of Bias tool for RCTs

| **Study** | **Random sequence generation** | **Allocation concealment** | **Blinding of participants and personnel** | **Blinding of outcome assessment** | **Incomplete outcome data** | **Selective reporting** | **Other bias** |
| --- | --- | --- | --- | --- | --- | --- | --- |
| **Logovinsky et al.** | **Unclear** | **Unclear** | **Low** | **Unclear** | **Low** | **Low** | **Low** |
| **Mcade et al.** | **High** | **High** | **Low** | **Unclear** | **Unclear** | **Low** | **High** |
| **Swanson et al.** | **Unclear** | **Unclear** | **Low** | **Unclear** | **High** | **Low** | **Low** |
| **Van Dyck et al.** | **Unclear** | **Unclear** | **Low** | **Low** | **Low** | **Low** | **Low** |

| Study | **Random sequence generation** | **Allocation concealment** | **Blinding of participants and personnel** | **Blinding of outcome assessment** | **Incomplete outcome data** | **Selective reporting** | **Other bias** |
| --- | --- | --- | --- | --- | --- | --- | --- |
| Logovinsky et al. | The clinical study used a multicenter double-blind randomized placebo-controlled design | The study does state that the study used a double-blind randomized placebo-controlled design, which suggests that some form of allocation concealment may have been used to ensure that neither the investigators nor the participants knew which treatment they were receiving. | Double blinded | The study does state that the study used a double-blind randomized placebo-controlled design, which suggests that some form of blinding may have been used to ensure that neither the investigators nor the participants knew which treatment they were receiving. | The study does state that all patients participating in the trial provided informed consent, and the authors acknowledge patients, caregivers, investigators, site personnel, and the BAN2401 project teams at Eisai and at BioArctic Neuroscience for their contributions. Additionally, the subject demographics in Table 1 are based on the total number of subjects with nonmissing values in the relevant treatment group, | The study does not provide any clear evidence of selective reporting in the clinical study. | The study does not provide any clear evidence of any other biase in the clinical study. |
| Mcade et al. | Of the 856 randomized subjects, 180 voluntarily enrolled into the OLE. Thus, subjects were not randomized by treatment and key disease characteristics into the OLE | It is mentioned that of the 856 randomized subjects, only 180 voluntarily enrolled in the OLE, which means that subjects were not randomized by treatment and key disease characteristics into the OLE. | Double-blinded | The authors did not mention directly that the assessors were unaware | total of 856 subjects were randomized into the study to receive either placebo (247 subjects) or lecanemab  (609 subjects). Of these, 552 subjects (177 placebo, 375  lecanemab) completed study 201 core. Of the 856 subjects randomized in study 201 core, 180 subjects entered  the OLE phase to receive lecanemab 10 mg/kg biweekly | The article does not provide any evidence of selective reporting | The OLE was started after a delay, resulting in a variable length gap period ranging from 9 to 59  months. |
| Swanson et al. | The randomization process in the trial involves an adaptive randomization method. Adaptive randomization is a procedure that adjusts the allocation probabilities based on the accumulating data in the trial. | There is no specific mention of how allocation concealment, a key factor in reducing selection bias, was implemented in the study. Therefore, the risk of bias for allocation concealment remains unclear. | The study is double blinded | The study does not provide explicit information about whether blinding of outcome assessment was employed in the BAN2401-G000-201 clinical trial. As a result, the risk of bias for blinding of outcome assessment is considered unclear. | At 12 months, the 10-mg/  kg biweekly ED90 dose showed a 64% probability to be better than placebo by 25% on ADCOMS, which missed  the 80% threshold for the primary outcome | The study does not provide any clear evidence of selective reporting in the clinical study. | The study does not provide any clear evidence of any other biase in the clinical study. |
| van Dyck et al. | The article does not explicitly mention the process of random sequence generation for the allocation of participants to the treatment and placebo groups. However, it does state that the participants were randomized in a 1:1 ratio to receive either lecanemab or placebo, and that the randomization was stratified by APOE ε4 carrier status and Clinical Dementia Rating (CDR) score at baseline. | The article does not explicitly mention the process of allocation concealment. However, it does state that the trial was double-blind, meaning that neither the participants nor the clinical assessment raters were aware of the treatment assignments. | Double blinded | the article states that the clinical assessment raters were unaware of the safety assessments and the trial-group assignments, indicating that outcome assessment was blinded. | Sensitivity analyses  across efficacy end points to assess the robustness  of the primary analysis to missing data included  rank analysis of covariance with imputation of  missing values | The article does not provide any evidence of selective reporting. The authors report the results of both primary and secondary endpoints, as well as sensitivity analyses, and provide detailed information on the methods used in the trial. The trial protocol is also available with the full text of the article at NEJM.org. Therefore, there is no indication of selective reporting in this study. | The article does not provide any evidence of other bias |
